# β-Glucan alleviates goal-directed behavioral deficits in mice infected with *Toxoplasma gondii*

**DOI:** 10.1186/s13071-023-05686-4

**Published:** 2023-02-13

**Authors:** Zeyu Cui, Yuying Gong, Xiaotong Luo, Niuyi Zheng, Shimin Tan, Shuxi Liu, Youwei Li, Qingling Wang, Fenfen Sun, Minmin Hu, Wei Pan, Xiaoying Yang

**Affiliations:** 1 Jiangsu Key Laboratory of Immunity and Metabolism, Department of Pathogen Biology and Immunology, Xuzhou Medical University, Xuzhou, 221004 Jiangsu China; 2 The Second Clinical Medical College, Xuzhou Medical University, Xuzhou, 221004 Jiangsu China; 3 Department of Anatomy, Basic Medical College, Xuzhou Medical University, Xuzhou, 221004 Jiangsu China; 4 The First Clinical Medical College, Xuzhou Medical University, Xuzhou, 221004 Jiangsu China; 5 Department of Pathology, Basic Medical College, Xuzhou Medical University, Xuzhou, 221004 Jiangsu China

**Keywords:** *Toxoplasma gondii*, β-Glucan, Prefrontal cortex, Cognitive impairment, Neuroinflammation

## Abstract

**Background:**

*Toxoplasma gondii* (*T. gondii*) is a neuroinvasive parasite causing neuroinflammation, which in turn is associated with a higher risk for several psycho-behavioral disorders. There is an urgent need to identify drugs capable of improving cognitive deficits induced by *T. gondii* infection. β-Glucan, an active ingredient in mushrooms, could significantly enhance immunity. However, the effects of β-glucan against neuroinflammation and cognitive decline induced by *T. gondii* infection remain unknown. The present study aimed to investigate the neuroprotective effect of β-glucan on goal-directed behavior of mice chronically infected by *T. gondii* Wh6 strain.

**Methods:**

A mice model of chronic *T. gondii* Wh6 infection was established by infecting mice by oral gavage with 10 cysts of *T. gondii* Wh6. Intraperitoneal injection of β-glucan was manipulated 2 weeks before *T. gondii* infection. Performance of the infected mice on the Y-maze test and temporal order memory (TOM) test was used to assess the goal-directed behavior. Golgi-Cox staining, transmission electron microscopy, immunofluorescence, real-time PCR and western blot assays were used to detect prefrontal cortex-associated pathological change and neuroinflammation.

**Results:**

The administration of β-glucan significantly prevented *T. gondii* Wh6-induced goal-directed behavioral impairment as assessed behaviorally by the Y-maze test and TOM test. In the prefrontal cortex, β-glucan was able to counter *T. gondii* Wh6-induced degeneration of neurites, impairment of synaptic ultrastructure and decrease of pre- and postsynaptic protein levels. Also, β-glucan significantly prevented the hyperactivation of pro-inflammatory microglia and astrocytes, as well as the upregulation of proinflammatory cytokines caused by chronic *T. gondii* Wh6 infection.

**Conclusions:**

This study revealed that β-glucan prevents goal-directed behavioral impairment induced by chronic *T. gondii* infection in mice. These findings suggest that β-glucan may be an effective drug candidate to prevent *T. gondii-*associated psycho-behavioral disorders including goal-directed behavioral injury.

**Graphical Abstract:**

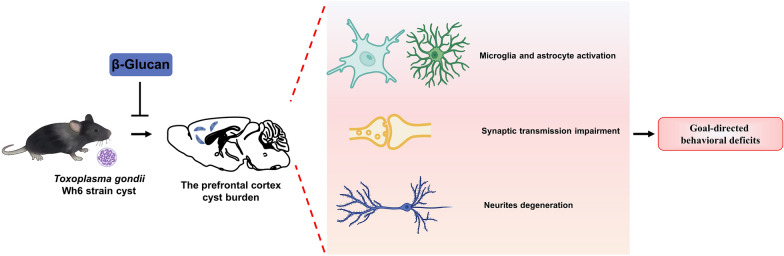

## Background

*Toxoplasma gondii* (*T. gondii*) is a central nervous system (CNS)-invasive protozoan parasite with a broad host range that infects nearly 30% of the global human population [1]. Upon infecting its host, *T. gondii* transforms into the replicative stage, spreads throughout the whole body and then quickly converts into the quiescent stage that forms cysts in host tissues, with a particular high accumulation of cysts in neurons of the CNS, establishing chronic infection for life. Generally, chronic *T. gondii* infections are asymptomatic [2]. However, compelling evidence links chronic *T. gondii* infection with psycho-behavioral disorders, such as Alzheimer's disease (AD), and Parkinson's disease (PD), that are characterized by cognitive dysfunction [3, 4]. For example, it has been reported that seropositive individuals infected with *T. gondii* exhibit subclinical alteration of behavior and a higher risk of PD [5] and that mice suffering from toxoplasmosis exhibit signs of AD [6]. Notably, most previous efforts to ameliorate the behavioral alterations caused by *T. gondii* infection have failed. Therefore, it is urgent to identify a new therapeutic strategy for the psycho-behavioral disorders induced by *T. gondii*.

The prefrontal cortex (PFC), which covers the anterior end of the brain, is an important brain region for regulating goal-directed behavior [7–9]. It is known that many psycho-behavioral disorders are characterized by various degrees of damage in the PFC [10–12]. Notably, in a recent randomized controlled trial, elderly *T. gondii*-seropositive individuals showed delaying processes of attentional allocation and disengagement [13]. In addition, in rodent models of chronic *T. gondii* infection, higher cyst density, neuroinflammation and neuronal degeneration were found in the PFC of mice [14, 15]. Another study reported that reduction of neuroinflammation could reverse the behavioral changes, such as hyperactivity, caused by latent toxoplasmosis [16]. Therefore, it would appear that remodeling neuroinflammation and neuronal degeneration is an effective approach to improve *T. gondii*-induced goal-directed behavioral disorders.

β-Glucan is an immunomodulating polysaccharide extracted from the *Lentinula edodes*, a cultivated edible and medicinal mushroom widely distributed throughout Southeast Asia [17]. As the main bioactive component of *L. edodes*, β-glucan could enhance host immune response and play a defensive role against pathogen invasion [18]. For example, it has been reported that β-glucan has the capability to protect the host from *Leishmania donovani* infection [19]. It has also been reported that β-glucan in combination with spiramycin can inhibit acute *T. gondii* infection [20, 21]. Interestingly, β-glucan can also reduce the secretion of the inflammatory factors interleukin (IL)-1β and IL-6 [22]. Previously, we demonstrated that dietary β-glucan can improve the steatohepatitis and cognitive impairment in high-fat diet-induced obese mice via remodeling of gut microbiota [23, 24]. Recently, another study reported that intraperitoneal injection of β-glucan induces immune training of microglia in mice [25]. However, it remains unknown if the intraperitoneal injection of β-glucan can improve goal-directed behavioral disorders caused by chronic *T. gondii* infection.

In this study, we hypothesized that intraperitoneal injection of β-glucan could ameliorate goal-directed behavior in mice chronically infected with *T. gondii*. Therefore, we infected mice with the Wh6 strain (a low virulence *T. gondii* strain in China) to induced goal-directed behavioral impairment [26]. We then evaluated the neuroprotective effects of β-glucan in the infected model. Specifically, the effects of β-glucan on PFC-related cognitive functions were evaluated based on performance on the Y-maze test and temporal order memory (TOM) test. Moreover, Transmission electron microscopy, Golgi-Cox staining and immunofluorescence were used to characterize the synaptic ultrastructure, neuronal dendrites and neuroinflammation, respectively, in the PFC of mice.

## Methods

### Animal care and treatment

C57BL/6J mice aged 7 weeks were obtained from the Experimental Animal Center of Xuzhou Medical University (Xuzhou, China; permit: SCXK (Su) 2020–0048). The mice were kept in cages (5 mice per cage) under environmentally controlled conditions (12/12-h light/dark cycle and 22 °C) and had unrestricted access to water and food. After 1 week of acclimatization, the mice were randomly divided into four groups (*n* = 10 mice per group): (i) mice receiving phosphate buffer saline (PBS) by oral gavage, as a vehicle control (NC) group; (ii) mice injected intraperitoneally with β-glucan solution (50 mg/kg body weight/mouse; Sigma-Aldrich, St. Louis, MO, USA) 2 weeks prior to PBS gavage (NB group); (iii) mice infected with 10 cysts of *T. gondii* Wh6 strain by oral gavage (*T. gondii* infection [TG]); (iv) mice injected intraperitoneally with β-glucan solution 2 weeks prior to *T. gondii* infection (β-glucan treated [TGB] group). The behavior tests (Y-maze and TOM tests) were performed at 4 weeks post-*T. gondii* infection, as detailed in the following sections, and all mice were euthanized 4 days after the behavioral experiment. Brain tissues of the sacrificed mice were dissected and frozen at − 80 °C until further analyses.

All experiments were performed according to methods approved by the ethics committee of Xuzhou Medical University. Each experiment was repeated three times independently.

### Animal model for *T. gondii* Wh6 infection

*Toxoplasma gondii* Wh6 strain was used in the present study (see [27] for more details). Ten brain cysts in 200 µl of PBS were gavaged into C57BL/6 J mice. Four weeks later, brain tissues of the infected mice were collected and homogenized in 1 ml of PBS. The cysts were counted microscopically. Ten brain cysts were gavaged into C57BL/6 J mice to establish mouse model of chronic *T. gondii* infection as previously reported [28].

### Y-Maze test

The Y-maze test was performed to measure spatial working retention based on procedures described previously [29]. In brief, the arms of the Y-maze were labeled with different pictures after acclimatization of the mice. Each mouse was put in the center of the maze and allowed to freely explore the maze for 8 min. The number of all arm entries and spontaneous alternations were recorded. A spontaneous alternation was defined as the mouse entering all three arms consecutively (i.e. in the order one, two, three, two, one, three and three, two, one, but not in the order three, one, three and two, one, one). The alternation triplet (%) was counted using the following formula: [number of spontaneous alternations/(number of entries to all arms entries-2) × 100].

### TOM test

Temporal order memory is an ability that involves maintaining the order in which events or items are experienced over time. This ability can be demonstrated by the ability to recall the order of previously experienced events/items or by the ability to distinguish which of two events/items was encountered most recently (or less recently). Animal experiments have shown that animals are able to distinguish between old and recent objects in a test delayed by 24 h. Even though there is only a short interval (1 h) between the old object and the most recent object at the most recent time, animals with good temporal order memory would spend significantly more time exploring the old object than the new object [30]. The TOM test was carried out to evaluate recognition memory processes according to methods described previously [31]. Briefly, the test consisted of three stages: two sample stages and one test stage. One day before the test, each mouse was placed in behavioral testing room for 60 min to acclimatize to the environments. During the two sample stages, each mouse explored two identical objects freely for 4 min; the objects between two sample stages were diverse, with object A for sample stage one and object B for sample stage two. The time intervals between each sample stage was 60 min. The test stage was started 120 min later. For the test stage, one object from the sample stage one (object A, old object) and another object from the sample stage two (object B, recent object) were presented at the same time, and each mouse was allowed to explore the open field freely for 3 min. The discrimination index (%) was calculated as follows: (old object exploration time − recent object exploration time)/(total exploration time) × 100. Intact temporal order memory was considered if the mouse used a longer time to explore the old object than the recent object.

### Transmission electron microscope

Following the cardiac perfusion with saline, the PFC region of the brain was rapidly dissected and fixed in glutaraldehyde for 24 h. The fixed PFC tissues of each experimental group (NC, NB, TG, TGB) were cut into thin sections, and the sections were fixed in 2.5% glutaraldehyde overnight at 4 °C. After three washes in PBS, the fixed sections were post-fixed with 1% osmium tetroxide, then stained with 2% uranyl acetate, dehydrated in a series of increasing concentrations of ethanol and acetone and embedded in epoxy resin. Finally, the sections were cut into 70-nm-thick slices on a ultramicrotome, and the slices were stained with 4% uranyl acetate and 0.5% lead citrate after being collected on copper grids. The ultrastructure of synapses in the PFC was observed by transmission electron microscopy (TEM) (FEI Company, Hillsboro, OR, USA), and the morphometrics of synapses were detected. The postsynaptic density (PSD), the width of synaptic clefts, the curvature of the synaptic interface and the length of the activated zone were determined using Image J software (version 1.53n; https://imagej.nih.gov/ij/).

### Golgi-Cox staining and image analysis

Variations in neuronal morphology were analyzed by Golgi-Cox staining using the FD Rapid Golgi Stain Kit (Nanjing Well-Offex Biotechnology Co., Ltd., Najing, China; catalog number: PK401) as previously described [32]. In brief, the brains of mice were dissected and impregnated with a mixture of solution A and solution B and then stored at room temperature for 14 days in the darkness, with replacement of the solution at 48-h intervals. The brain tissues were then immersed in solution C for 5 days, and sectioned (200-μm-thick slices) using a vibratome. The sections were mounted on gelatinized slides, stained with a mixture of solution D and solution E, dehydrated by passage through a series of increasing concentrations of alcohol, hyalinized with xylene and finally covered with Permount. Images were taken using a digital camera attached to a microscope (Olympus Corp., Tokyo, Japan). Investigators blind to experimental design randomly chose dendritic shafts and spines of pyramidal neurons from layers II/III of the PFC for analysis. The Neuron J plugin of ImageJ software was used to analyze the morphological data, including the total neurite length of the neuron, the length of each neurite and the number of neurites per neuron. The spine density of dendritic spines was estimated by counting the number of spines along a section of shaft (10-μm length on a 30- to 50-μm-long segment of a distal branch) using the Cell Counter plugin of ImageJ software. A Sholl analysis was also performed to evaluate dendritic complexity by using the Sholl plugin of ImageJ software [33]. Images of Golgi-stained neurons were overlaid by concentric circles of increasing diameters (10-μm increases) around the soma (10–300 μm). The number of neurites crossing each circle was counted manually. The number of intersections between a circle of a given radius and neurites was assessed, and the following indicators were calculated: (i) sum of intersections; and (ii) maximum intersection distance.

### Immunofluorescence

Immunofluorescence was carried out according to methods described previously [34]. In brief, dissected brains were fixed in 4% paraformaldehyde overnight, dehydrated in 30% sucrose in PBS and stored in a − 20 °C freezer. All sections for immunofluorescence were cut into 3-μm-thick slices on a rotary microtome (RM2016; Leica Biosystems, Wetzlar, Germany). The brain slices were then blocked with 5% bovine serum albumin (Servicebio Technology Co., Ltd., Ghent, Belgium; catalog number GC305010) for 30 min at room temperature, following which they were incubated with the primary antibody anti-Iba1 (Abcam, Cambridge, UK; catalog number Ab178847) or anti-glial fibrillary acidic protein (GFAP; Cell Signaling Technology, Danvers, MA, USA; catalog number 3670) or anti-IL-6 (Servicebio Technology Co., Ltd.; catalog number GB11117) at 4 °C overnight. The sections were then incubated with horseradish peroxidase (HRP)-inked goat anti-rabbit immunoglobulin G (IgG) secondary antibody (Servicebio Technology Co., Ltd.; catalog number GB23303) or Cy3 conjugated goat anti-mouse IgG secondary antibody (Servicebio Technology Co., Ltd.; catalog number GB21303) for 1 h at room temperature. After washing, the sections were incubated with fluorescein isothiocyanate–tyramide signal amplification (FITC-TSA) reagent (Servicebio Technology Co., Ltd.; catalog number G1222) for 10 min at room temperature, placed in a retrieval box containing EDTA antigen retrieval buffer (Servicebio Technology Co., Ltd.; catalog number G1206) and heated in a microwave to facilitate removal of de-bound primary and secondary antibodies. The antibody rabbit anti-Iba1 (Abcam; catalog number Ab178847) or anti-GFAP (Cell Signaling Technology; catalog number 3670) was dropped onto the slices and the slices incubated at 4 °C overnight. The sections were then incubated with Cy3-conjugated goat anti-rabbit IgG secondary antibody (Servicebio Technology Co., Ltd.; catalog number GB21303) for 50 min at room temperature. Finally, the slices were stained with DAPI (Servicebio Technology Co., Ltd.; catalog number G1012). The morphology and the percentage of IL-6-positive (IL-6^+^) microglia (Iba1-positive [IBa1^+^] cells) and astrocytes (GFAP-positive [GFAP^+^] cells) in the PFC were then captured using a microscope (Eclipse C1; Nikon Corp., Tokyo, Japan) and quantified using Image J software. The formula for circularity is 4π × area/perimeter^2^, with a value of 1.0 indicating a perfect circle; the closer the value approaches 0.0, the more it indicates an increasingly elongated shape. Values may not be valid for very small particles. The formula for solidity is area/convex area [35].

### Western blotting

Western blot assays were performed as described previously [34]. Briefly, proteins were extracted from PFC in ice-cold RIPA lysis buffer containing EDTA, protease inhibitor cocktail (Beyotime Biotech, Shanghai, China) and 1 mM phenylmethylsulfonyl fluoride (PMSF; Beyotime Biotech). The protein concentrations was measured by BCA assay (Beyotime Biotech). Samples of 20–40 μg proteins were resolved by electrophoresis in a 10% sodium dodecyl sulfate–polyacrylamide gel (SDS-PAGE), and the products were electrotransferred onto polyvinylidene difluoride membranes. Western blot assays were conducted by using primary antibodies, including anti-BDNF (Alomone Labs, Jerusalem, Israel; catalog number ANT-010), anti-PSD95 (Cell Signaling Technology; catalog number 3450), anti-Synaptophysin [YE269] (Abcam; catalog number ab32127) and anti-β-actin (Cell Signaling Technology; catalog number #4967). Secondary antibody was HRP-inked anti-rabbit IgG (Cell Signaling Technology; catalog number 7074). Immunodetection was made using Clarity™ ECL Western Blot Substrate (Bio-Rad Laboratories, Hercules, CA, USA and the images were captured using the ChemiDoc Touch System (Bio-Rad Laboratories).

### RNA extraction and real-time PCR

RNA extraction and real-time reverse transcription PCR (RT-PCR) were performed based on methods previously described [24]. Briefly, total RNA was extracted from PFC homogenized in TRIzol (Thermo Fisher Scientific, Waltham, MA, USA). A 1 μg sample of RNA subjected to reverse-transcription to complementary DNA (cDNA) using a High-Capacity cDNA Reverse Transcription Kit (Takara, Dalian, China). RT-PCR assays were then performed using the SYBR GREEN Master Mix (Takara) and detected on a real-time PCR detection system (Bio-Rad Laboratories). The relative messenger RNA (mRNA) levels were calculated using the 2^−ΔΔCt^ method and normalized by β-actin mRNA levels. The primers (and their sequences) were: mTNFα–forward (F) (CTTGTTGCCTCCTCTTTTGCTTA); mTNFα–reverse (R) (CTTTATTTCTCTCAATGACCCGTAG); mIL-6–forward (F) (CTGCTCATTCACGAAAAGGGA); mIL-6–reverse (R) (TCACAGAAGGAGTGGCTAAGGACC); mβ-actin–forward (F) (CGTGGGCCGCCCTAGGCACCA); mβ-actin–reverse (R) (TTGGCCTTAGGGTTCAGGGGGG).

### *Toxoplasma gondii* cyst counts

Cyst counts were quantified using the method reported previously [36]. In brief, brain tissues were crushed in 1 ml PBS, and the cysts in 10 μl brain suspension were counted under a light microscopy (magnification: 20×). The number of cysts was counted in a blind manner to evaluate the total cyst burden in the brain tissue. The counting process for each mouse was repeated 4 times.

### Statistical analysis

Data are presented as the mean ± standard error of the mean. Statistical analysis was performed using SPSS version 20 (SPSS-IBM Corp., Armonk, NY, USA). All data were tested for normality by applying the Shapiro–Wilk normality test. If normality was given, comparisons between two groups were done by using the unpaired t-test, while comparisons between multiple groups were conducted by one-way analysis of variance followed by the post hoc Tukey’s test. A *P* value < 0.05 was considered to indicate statistical significance.

## Results

### β-Glucan prevented the goal-directed behavioral impairment caused by *T. gondii* Wh6 infection in mice

**Fig. 1:**
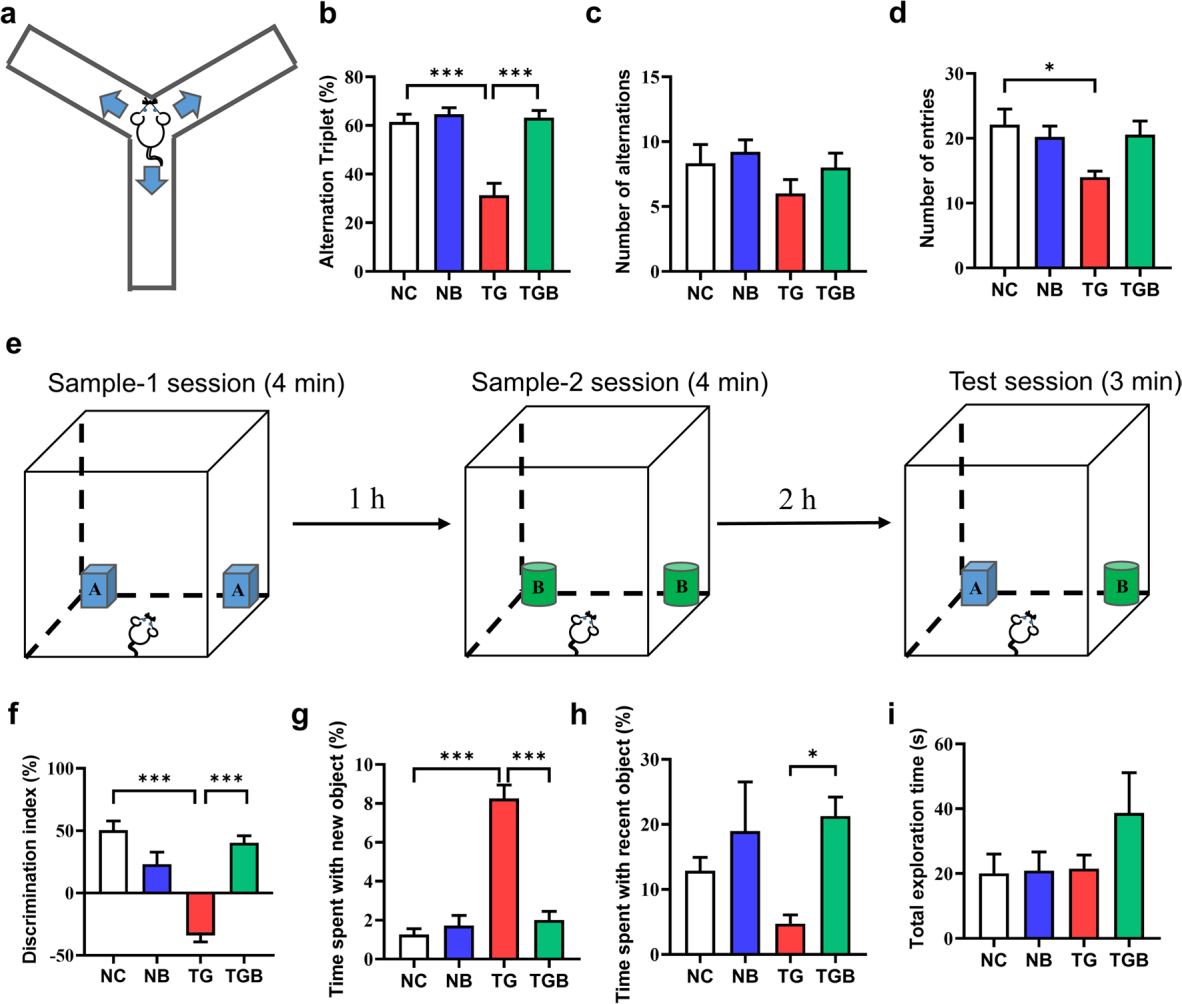
β-Glucan administration prevented chronic *Toxoplasma gondii* Wh6-induced goal-directed behavioral impairment in mice. Ten brain cysts in 200 µl of phosphate-buffered saline (PBS) were gavaged into C57BL/6J mice to establish the *T. gondii* Wh6 infection model. Four weeks after infection, the behavior performance of mice were evaluated by the Y-maze and temporal order memory (TOM) tests. **a–d** The aim of the Y-maze test was to examine the spatial memory of mice: **a** Y-maze test device diagram, **b** percentage of alternation triplet in the Y-maze test, **c** number of alternation in the Y-maze test, **d** number of entries in the Y-maze test. **e–i** The TOM test was used to appraise the temporal order memory of mice: **e** TOM test flow chart, **f** discrimination index in TOM, **g** percentage of time spent exploring the new object in TOM, **h** percentage of time spent exploring the old object in TOM, **i** total exploration time in TOM. Values are presented as the mean ± standard error of the mean (SEM). Asterisks indicate a statistically significant difference at *P < 0.05, ***P < 0.001, according to one-way analysis of variance (ANOVA) followed by Tukey’s test for multiple comparisons. NC, mice gavaged with PBS as a vehicle control (normal control); NB group, mice intraperitoneally injected with β-glucan solution 2 weeks prior to PBS gavage; TG, mice gavaged with 10 cysts of *T. gondii* Wh6 strain (*T. gondii-*infected group); TGB, mice intraperitoneally injected with β-glucan solution 2 weeks prior to *T. gondii* infection (β-glucan-treated *T. gondii-*infected group)

To assess whether β-glucan treatment could prevent abnormal goal-directed behavior induced by *T. gondii* infection, we evaluated the behavior of the mice in performing the Y-maze and TOM tests, which enables PFC-dependent spatial working and recognition memory of daily living to be assessed [29, 37]. The schematic timeline for behavior tests is shown in Fig. 1a. In the Y-maze test, the ratio of spontaneous alteration in the TG group (*T. gondii-*infected group) was clearly lower than that in the NC group (control group gavaged with PBS only), while β-glucan significantly increased the alternation triplet of the TG group (*F*_(3, 32)_ = 20.19, *P* < 0.0001; Fig. 1b). The difference in alternation triplet was not due to the variant general activity because the number of alternations and entries did not markedly differ among the four groups (*F*_(3, 32)_ = 1.383, *P* = 0.2656, Fig. 1c;* F*_(3, 32)_ = 3.683, *P* = 0.0219, Fig. 1d). In the TOM test, recognition memory was negatively affected in the TG group, as demonstrated by *T. gondii* infection significantly reducing the discrimination index compared with that of the NC group, while β-glucan significantly increased the novel object discrimination index of the TG group (*F*_(3, 32)_ = 27.77, *P* < 0.0001; Fig. 1e, f). In more detail, mice in the TG group spent more time in recognizing new objects, and this behavior was improved by β-glucan treatment (*F*_(3, 32)_ = 41.50, *P* < 0.0001; Fig. 1g). The time spent with the most recent objects and the total exploration time of objects during the test stage were similar among these groups (Fig. 1h, i). Overall, these results indicate that goal-directed behavioral deficits caused by *T. gondii* Wh6 infection are preventable by β-glucan treatment.

### β-Glucan mitigated neurite impairment in the PFC of* T. gondii* Wh6-infected mice

**Fig. 2:**
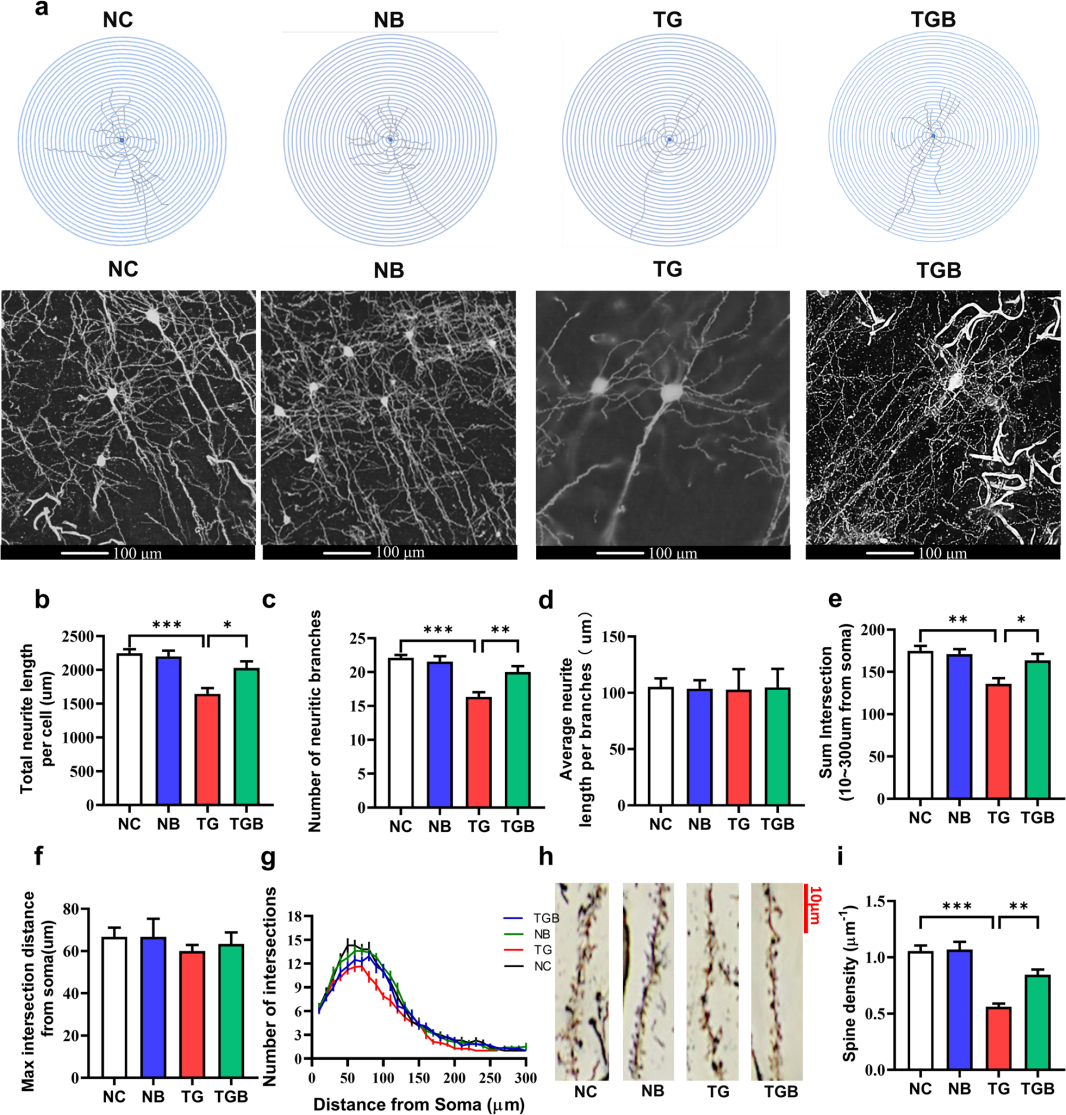
β-Glucan administration ameliorated neurite degeneration and the decrease in dendritic spine density in the prefrontal cortex (PFC) of chronic *T. gondii* Wh6-infected mice. Variations in neuronal morphology were analyzed by Golgi-Cox staining. **a** Representative images of pyramidal neurons in the PFC of mice. Scale bars: 100 μm. **b** Total neurite length per cell. **c** Number of neurite branches. **d** Average neurite length per branch. **e** The sum intersection of neuron.** f** The max intersection distance from soma (*n* = 10/group). **g** Sholl analysis of dendritic branching complexity of pyramidal neurons in the PFC of mice. **h** Representative images of dendritic spines of neurons in the PFC. Scale bars: 10 μm. The sum intersection of neurons. **i** Spine density. Sholl analysis of dendritic branching complexity of pyramidal neurons in the PFC of mice. Values are presented as the mean ± SEM. Asterisks indicate a statistically significant difference at *P < 0.05, ** *P* < 0.01, *** *P* < 0.001, according to one-way ANOVA followed by Tukey’s test for multiple comparisons

Since improved goal-directed behavior was observed following β-glucan administration, we therefore looked at the effects of β-glucan on neurite impairment of the PFC in *T. gondii* Wh6-infected mice by examining the structure of neurites in the PFC after Golgi-Cox staining. A significant decrease in the total neurite length per cell and in the number of neurite branches were observed after *T. gondii* Wh6 infection, while the injection of β-glucan reversed these changes (*F*_(3, 32)_ = 10.56, *P* < 0.0001; Fig. 2b; *F*_(3, 32)_ = 13.08, *P* < 0.0001; Fig. 2c). There were no significant changes in the average neurite length per branch (*F*_(3, 32)_ = 0.007356, *P* = 0.9991; Fig. 2d). Also, Sholl analysis showed that compared with mice in the NB group, mice in the TG group had reduced dendritic complexity and synaptic spines morphology in the PFC by reducing the sum number of dendritic intersections, the distance of the maximum intersections from the soma and spine density, while these abnormalities were improved to some degree by β-glucan (*F*_(3, 32)_ = 6.912, *P* = 0.001, Fig. 2e;* F*_(3, 32)_ = 0.3056, *P* = 0.8212, Fig. 2f;* F*_(3, 32)_ = 22.03, *P* < 0.0001, Fig. 2i). Collectively, these results indicate that β-glucan was able to prevent neurite degeneration and decreased neuronal complexity in the PFC induced by *T. gondii* Wh6 infection.

### β-Glucan ameliorated synaptic ultrastructural damage in the PFC of* T. gondii* Wh6-infected mice

**Fig. 3:**
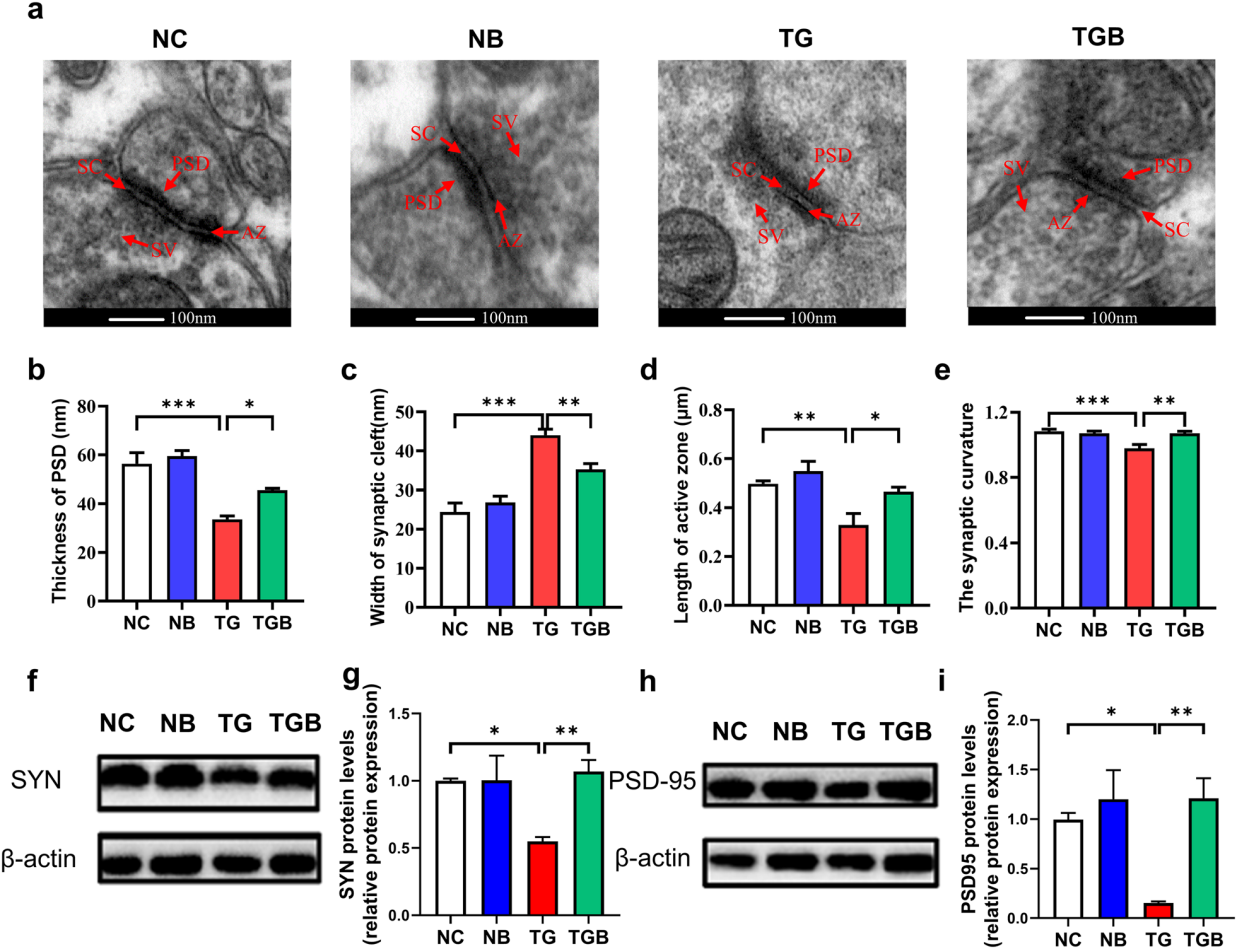
β-Glucan administration improved synaptic ultrastructural impairment in the PFC of chronic *T. gondii* Wh6-infected mice. **a–e** Synaptic ultrastructure was analyzed by transmission electron microscope: **a** Representative images of synaptic ultrastructure in the PFC of mice (scale bars: 100 nm), **b-e** the statistical analysis of the synaptic ultrastructure associated indexes: **b** thickness of PSD, **c** width of synaptic cleft, **d** length of active zone, **e** synaptic curvature, **f–i** Western blot assays were performed to detect SYN and PSD-95 protein levels: **f, g** SYN protein levels in the PFC of mice; **h**, **i** PSD-95 protein levels in the PFC of mice. Values are presented as the mean ± SEM. Asterisks indicate a statistically significant difference at *P < 0.05, ** *P* < 0.01, *** *P* < 0.001, according to one-way ANOVA followed by Tukey’s test for multiple comparisons. AZ, Active zone; PSD-95, postsynaptic density protein 95; SC, synaptic cleft, SV, synaptic vesicle; SYN, synaptophysin

Synaptic ultrastructure and synaptic plasticity are strongly associated with spatial working and recognition memory [38]. We therefore examined the effects of β-glucan on synaptic ultrastructure and the expression of synaptic plasticity proteins. The ultrastructure of synapses in the PFC was analyzed by TEM. We found that *T. gondii* Wh6 infection reduced the thickness of PSD, shortened the length of the active zone, decreased synaptic curvature and increased the width of the synaptic cleft. Notably, compared with the TG group, these pathogenic changes were attenuated following β-glucan administration, with mice in these latter groups exhibiting thicker PSD, longer active zones, higher synaptic curvature and reduced width of the synaptic cleft (*F*_(3, 32)_ = 15.58, *P* < 0.0001, Fig. 3b; *F*_(3, 32)_ = 25.61, *P* < 0.0001, Fig. 3c; *F*_(3, 32)_ = 8.151, *P* = 0.0004, Fig. 3d; *F*_(3, 32)_ = 9.129, *P* = 0.0002, Fig. 3e). Also, the expression of the plasticity proteins synaptophysin (SYN) and postsynaptic density protein 95 (PSD-95) in the PFC was reduced in the TG group; however, none of these changes were found in the TGB group (*F*_(3, 20)_ = 3.888, *P* = 0.0244, Fig. 3g;* F*_(3, 20)_ = 7.570, *P* = 0.0014, Fig. 3i). In summary, these results suggest that β-glucan could ameliorate synaptic ultrastructure and synaptic plasticity of the PFC, thereby preventing PFC-associated cognitive decline caused by *T. gondii* Wh6 infection.

### β-Glucan suppressed microglia activation in the PFC of *T. gondii* Wh6-infected mice

**Fig. 4:**
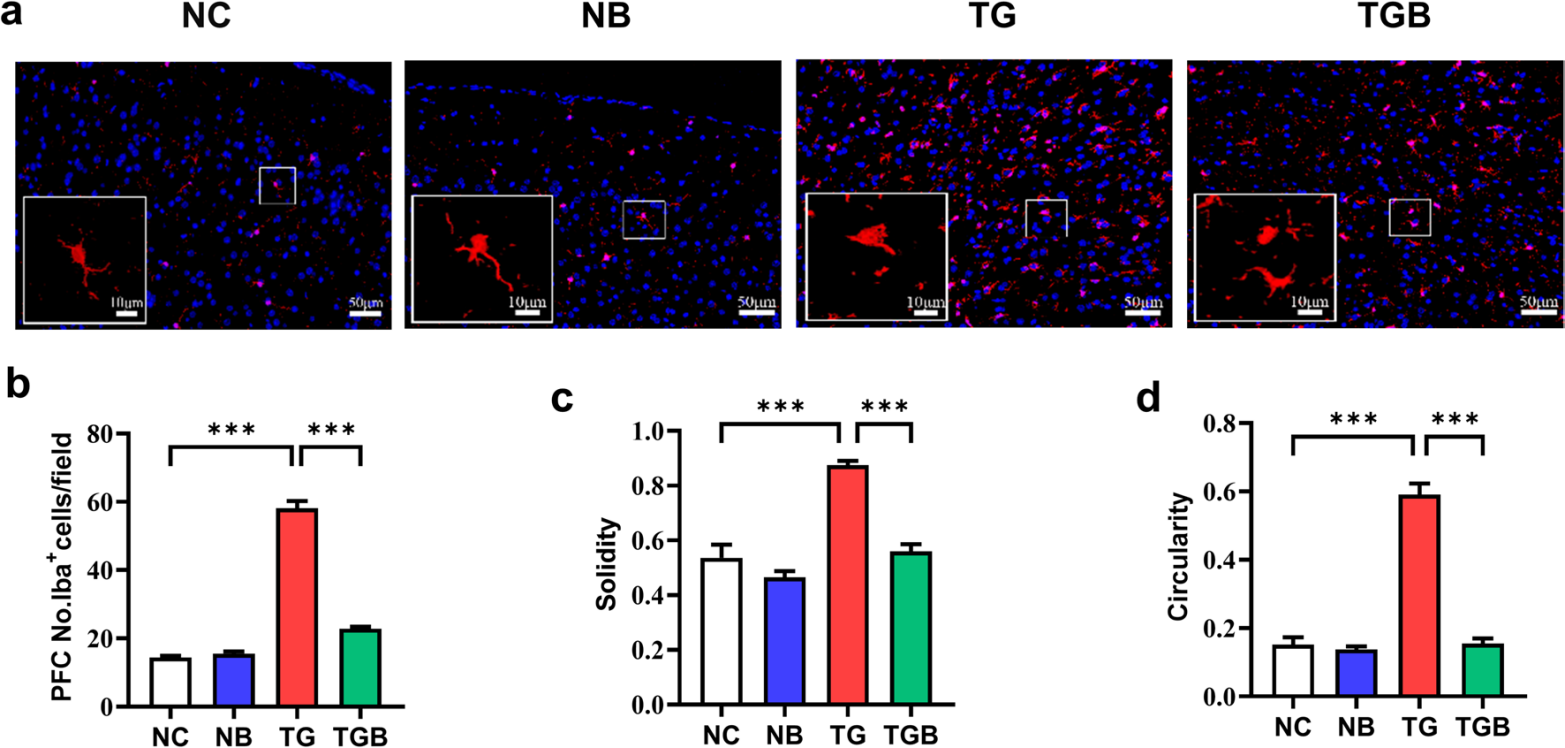
β-Glucan administration inhibited the hyperactivation of microglia in the PFC of chronic *T. gondii* Wh6-infected mice. Immunofluorescent staining was carried out to detect the number and morphology of microglia in the PFC of chronic *T. gondii* Wh6-infected mice. **a** Representative immunofluorescent staining of the Iba1-positive (Iba1^+^) cells of microglia in the PFC of mice (scale bar: 50 μm). Area in box in each image is enlarged in the respective inset in each image, marked with a solid line (scale bar: 10 μm). Solidity and circularity are used to describe the morphology of microglia. **b** Quantification of Iba1^+^ microglia in the PFC, **c** solidity of Iba1^+^ microglia in the PFC, **d** circularity of Iba1^+^ microglia in the PFC. Values are presented as the mean ± SEM. Asterisks indicate a statistically significant difference at *** *P* < 0.001, according to one-way ANOVA followed by Tukey’s test for multiple comparisons

Activation of microglia are critical in the process of neurodegeneration [39]. We therefore investigated the effects of β-glucan on microgliosis in the PFC of mice infected with *T. gondii* Wh6. Using Iba-1 as the immunofluorescent marker of microglia, we found that *T. gondii* infection significantly increased the number of microglia in the PFC, while β-glucan was clearly observed to decrease the number of microglia (*F*_(3, 36)_ = 303.7, *P* < 0.0001; Fig. 4b). Further analysis of the morphology of microglia revealed that the circularity and solidity of microglia were obviously increased in the PFC of the TG group compared with those of the NC, NB and TGB groups (*F*_(3, 36)_ = 35.51, *P* < 0.0001, Fig. 4c; *F*_(3, 36)_ = 106.4, *P* < 0.0001, Fig. 4d). These results suggest that microglial activation induced by *T. gondii* Wh6 infection could be mitigated by β-glucan supplementation.

### β-Glucan inhibited astrocyte activation in the PFC of *T. gondii* Wh6-infected mice

**Fig. 5:**
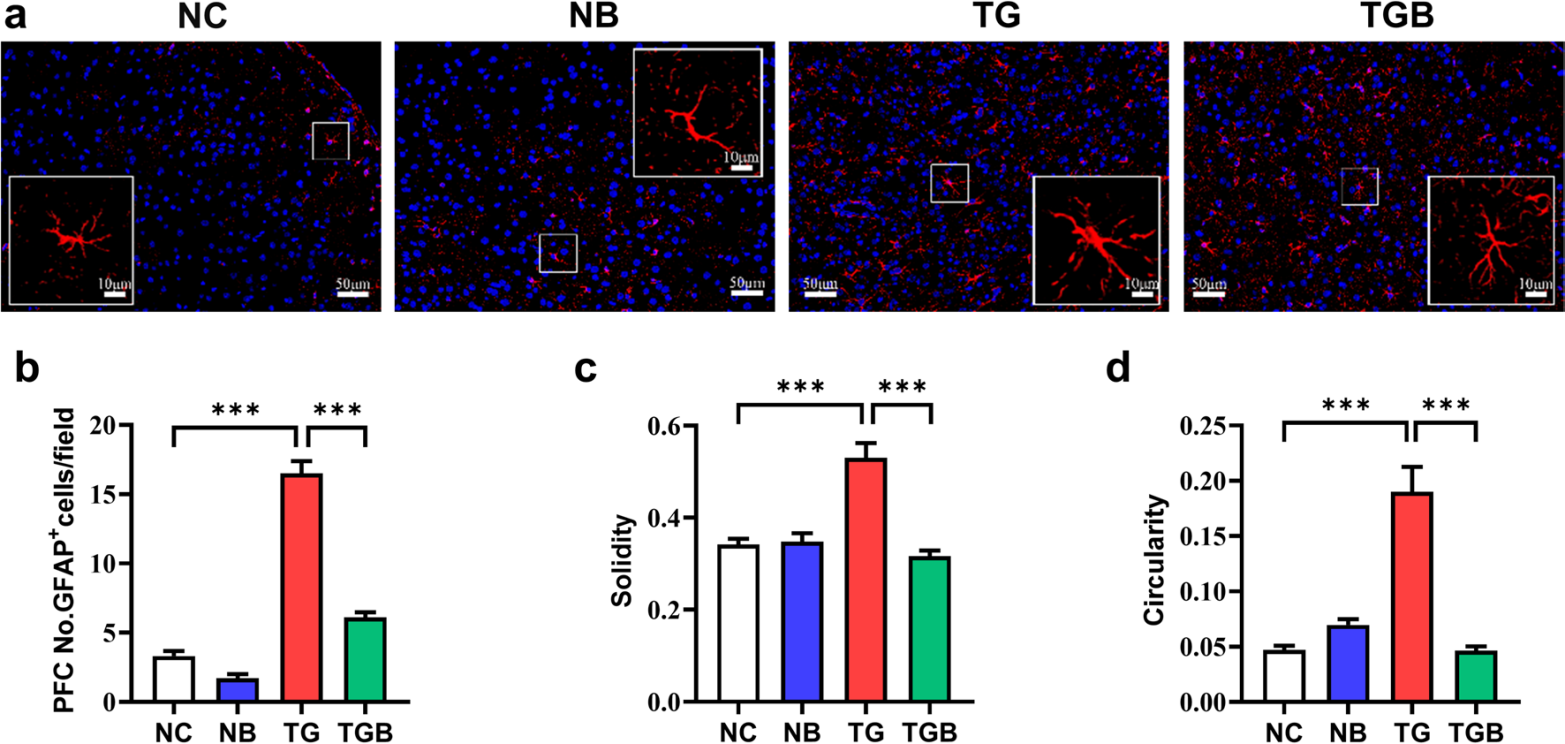
β-Glucan administration restrained the hyperactivation of astrocyte the PFC of chronic *T. gondii* Wh6-infected mice. Immunofluorescent staining was performed to detect the number and morphology of astrocytes in the PFC of chronic *T. gondii* Wh6-infected mice. **a** Representative immunofluorescence staining of GFAP-positive (GFAP^+^) astrocytes in the PFC of mice (scale bar: 50 μm). Area in box in each image is enlarged in the respective inset in each image, marked with a solid line (scale bar: 10 μm). Solidity and circularity are used to describe the morphology of astrocyte (*n* = 3 mice, 9–12 cells per mouse). **b** Quantification of GFAP^+^ astrocyte in the PFC. **c** Solidity of GFAP^+^ astrocyte in the PFC. **d** Circularity of GFAP^+^ astrocyte in the PFC. Asterisks indicate a statistically significant difference at *** *P* < 0.001, according to one-way ANOVA followed by Tukey’s test for multiple comparisons. GFAP, Glial fibrillary acidic protein

Abnormal activation of astrocytes is another risk factor for cognitive decline [40]. Therefore, we examined the effects of β-glucan on astrogliosis in the PFC of mice infected with *T. gondii* Wh6. Using GFAP as the immunofluorescent marker of astrocyte, we observed that the number of astrocytes in the PFC of mice in the TG group was markedly lower than that in the PFC of mice in the NC group, and that the number was restored by β-glucan supplement (*F*_(3, 36)_ = 153.8, *P* < 0.0001; Fig. 5b). We also analyzed the morphology of astrocytes in the PFC and found an enlarged cell body and fewer ramifications in the PFC of the TG group compared with the PFC of TGB group (*F*_(3, 36)_ = 23.37, *P* < 0.0001, Fig. 5c; *F*_(3, 36)_ = 33.34, *P* < 0.0001, Fig. 5d). These results suggest that β-glucan intervention could prevent astrocyte activation in the PFC of mice infected with *T. gondii* Wh6.

### β-Glucan restricted the expression of pro-inflammatory cytokines in the PFC of *T. gondii* Wh6-infected mice

**Fig. 6:**
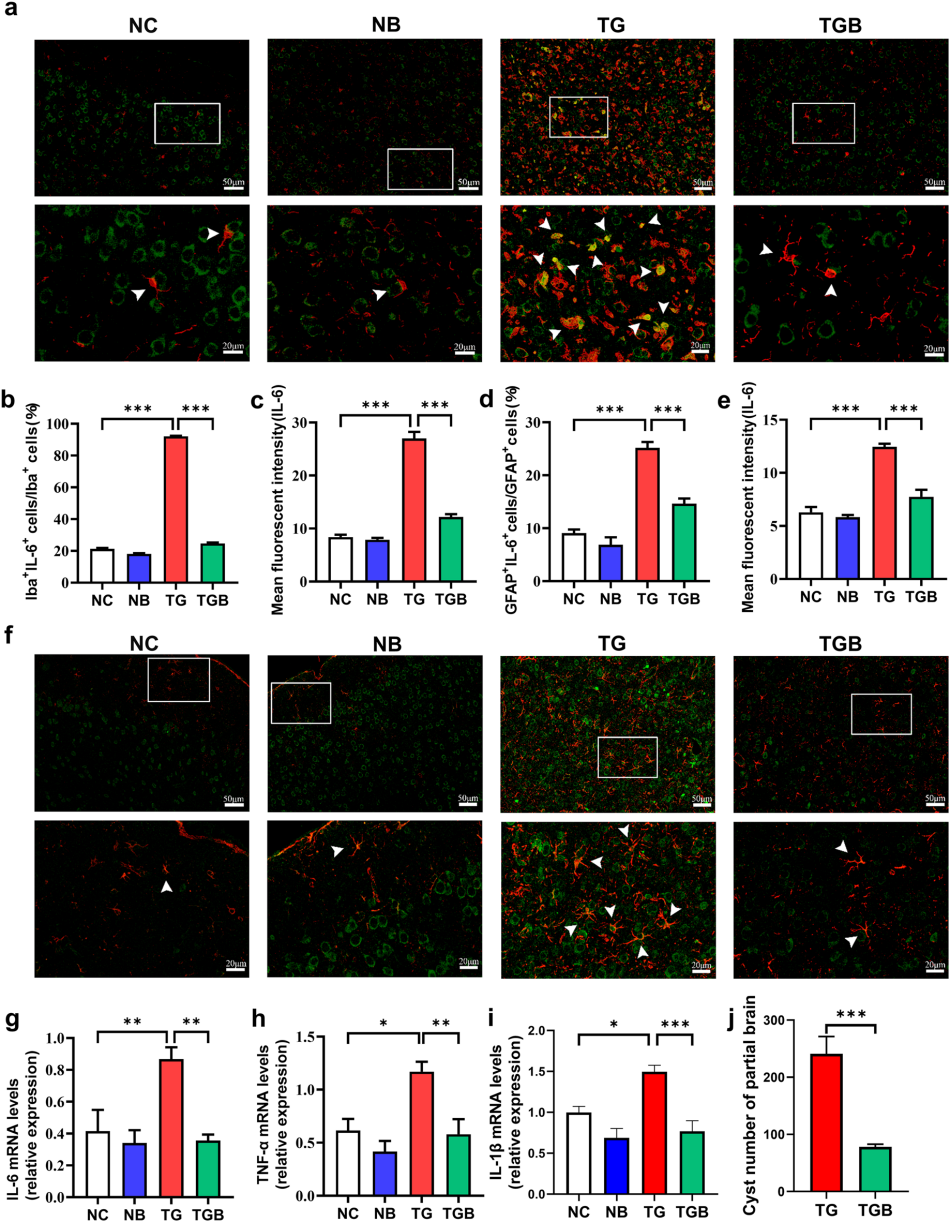
β-Glucan administration suppressed the expression of pro-inflammatory cytokines in the PFC of chronic *T. gondii* Wh6-infected mice. Double immunofluorescence staining was performed to detect the IL-6 expression in the microglia and astrocyte of chronic *T. gondii* Wh6-infected mice. **a** Representative double immunofluorescence staining of Iba1 (red) and IL-6 (green) in the PFC of mice. The white arrowheads point to Iba1^+^IL-6-positive (IL-6^+^) cells. Scale bar: 50 μm or 20 μm. **b** Percentage of Iba1^+^IL-6^+^ cells in Iba1^+^ cells in the PFC. **c** Quantification of the mean fluorescence intensity of IL-6^+^ cells in the PFC. **d** Percentage of GFAP^+^IL-6^+^ cells in GFAP^+^ cells in the PFC. **e** Quantification of the mean fluorescence intensity of GFAP^+^ cells.** f** Representative double immunofluorescence staining of GFAP (red) and IL-6 (green) in the PFC of mice. Scale bar: 50 μm or 20 μm. **g** Real-time reverse transcription PCR (RT-PCR) analysis of IL-6 mRNA expression in the PFC. **h** RT-PCR analysis of TNF-α mRNA expression in the PFC. **i** RT-PCR analysis of IL-1β mRNA expression of in the PFC. **j** Cyst numbers in the brain of chronic *T. gondii* Wh6-infected mice. Values are presented as the mean ± SEM. Asterisks indicate a statistically significant difference at *P < 0.05, ** *P* < 0.01, *** *P* < 0.001, according to one-way ANOVA followed by Tukey’s test for comparisons among four groups and the unpaired t-test for comparisons between two groups. IL, Interleukin; mRNA, messenger RNA; TNF-α, tumor necrosis factor alpha

Following our observation that β-glucan improved microglial and astrocyte activation, we examined the effects of β-glucan on pro-inflammatory cytokines. Using double immunofluorescent staining of Iba-1 and IL-6, we found that the average intensity of fluorescence in IL-6-positive (IL-6^+^) cells and the percentages of Iba1^+^IL-6^+^ cells in Iba1^+^ cells in the TG group were significantly higher than those in the NC group, while β-glucan significantly inhibited these alternations (*F*_(3, 36)_ = 4495,* P* < 0.0001, Fig. 6b;* F*_(3, 36)_ = 145.3,* P* < 0.0001, Fig. 6c). Similar results were found in the astrocytes of the PFC using double immunofluorescent staining of GFAP and IL-6 to evaluate IL-6 expression in astrocytes: higher mean fluorescence intensity of IL-6^+^ cells and percentages of GFAP^+^IL-6^+^ cells in GFAP^+^ cells in the PFC were found in the TG group than in other groups (*F*_(3, 36)_ = 58.65,* P* < 0.0001, Fig. 6d;* F*_(3, 36)_ = 45.79,* P* < 0.0001, Fig. 6e). In addition, β-glucan strikingly prevented the upregulation of IL-6, tumor necrosis factor alpha (TNF-α) and IL-1β mRNA expression in the PFC (*F*_(3, 24)_ = 8.641, *P* = 0.0005, Fig. 6g; *F*_(3, 24)_ = 5.388, *P* = 0.0056, Fig. 6h;* F*_(3, 24)_ = 8.560, *P* = 0.0005, Fig. 6i). We also found that the number of cysts in the PFC of *T. gondii-*infected mice was reduced by β-glucan intervention (*t*_(10)_ = 5.317, *P* = 0.0003; Fig. 6j). These findings indicate that β-glucan suppressed the neuroinflammation induced by *T. gondii* Wh6 infection.

## Discussion

Using the *T. gondii* chronic infection model established by infecting mice with 10 cysts of *T. gondii* Wh6, we demonstrated that chronic *T. gondii* Wh6 infection induced PFC-dependent abnormal goal-directed behavior, which could be prevented by β-glucan administration. To our knowledge, this is the first study showing that β-glucan could prevent *T. gondii* Wh6-induced goal-directed behavioral disorder via amelioration of neuron integrity, synaptic ultrastructure and neuroinflammation. Importantly, our finding on the benefits of β-glucan to cognitive function may provide a new therapeutic strategy for the treatment of neurodegenerative diseases caused by toxoplasmosis.

It has been reported that chronic *T. gondii* infection is closely linked with psycho-behavioral disorders, such as AD [41]. It is important to note that neurodegenerative diseases such as AD are often characterized by the development of neuroinflammation in the CNS, structural damage to neuronal structures and loss of messaging function within the brain, as well as damage to synaptic ultrastructure and synaptic dysfunction [42]. All of these signs and disorders been found in brains after chronic *T. gondii* infection, suggesting a high degree of pathological correlation between chronic toxoplasma infection and diseases such as AD [43]. The authors of a study involving a community-based sample reported that *T. gondii*-seropositive individuals displayed subclinical behavioral changes and had a higher risk of PD [5]. Most previously published studies used type II strains, which are the classical* Toxoplasma* strains, mainly prevalent in Europe, Africa and North America, to study the cognitive impairment induced by toxoplasmosis, since type II strains can develop into the latent phase of toxoplasmosis and form cysts in the brain [44]. In China, Chinese 1 genotype Wh6 strain is the dominant genotype among the* Toxoplasma* strains circulating in China, and is an unique genotype that distinguishes it from the classical type II strains, such as possessing both ROP16I/III and GRA15II effectors, lower virulence, a greater tendency to form cysts and cause latent infection and easier transmission among cats and humans [45]. Recently, the Wh6 strain has been reported to induce tau phosphorylation in mice, similar to the pathological characteristics of AD [27]. However, there is only limited information about the association of Wh6 infection and neurodegenerative diseases.

In the present study, we found that Wh6-infected mice exhibited abnormal goal-directed behavior, based on the Y-maze and temporal order memory tests (Fig. 1), suggesting that *T. gondii* Wh6 impairs cognitive function. There have been many efforts in past studies to mitigate abnormal behaviors induced by *T. gondii*, but most of them have been unsuccessful [16]. For example, diphenyl diselenide treatment can normalize acetylcholinesterase activity in the CNS of chronically infected mice, but it failed to reverse the behavioral deficits [46]. Importantly, in the present study, intraperitoneal administration of β-glucan significantly prevented *T. gondii* Wh6-induced goal-directed behavioral impairment. In addition, cyst burden in the brain has been linked with the development of psycho-behavioral disorders, although mice infected with a *T. gondii* strain incapable of forming cysts stilldisplay behavior change [16, 47]. Rodents with chronic *T. gondii* infection were found to develop cysts in the PFC [14, 15]. In the present study we also found that cysts formed in the PFC of mice infected with *T. gondii* Wh6; however, the number of cysts in the PFC of infected mice was reduced by the β-glucan intervention. The mechanism of how β-glucan decreases cyst formation should be further examined in future studies. Neuron apoptosis is another characteristic of AD. It has been reported that chronic Wh6 infection causes neuron apoptosis in mice [27], but a different study reported that *T. gondii* could inhibit the apoptotic pathway in host cells, which allows the parasite to keep its replicative niche [48]. The influence of β-glucan on neuron apoptosis needs to be examined further.

The PFC, a region associated with cognitive processing, spatial working and recognition memory, is very susceptible to *T. gondii* infection [12, 14, 15, 49, 50]. Neurite arborization and synaptic plasticity are essential for neuronal circuits and cognitive function [51, 52]. Chronic *T. gondii* infection has been found to induce neuronal degeneration and to damage the synaptic ultrastructure in the PFC of mice [14, 15, 53, 54]. Clément et al. showed that *T. gondii* infection disrupted the neuronal cytoskeleton and caused synaptic loss in the PFC of mice [55]. In the present study, using Golgi-Cox staining and TEM, we observed the neurite degeneration, the decreased neuronal complexity and the impaired synaptic transmission in the PFC of the TG group (Figs. 2, 3a–e). These results powerfully suggest that *T. gondii* Wh6 impairs neurite arborization and synaptic plasticity, resulting in cognitive impairment. Importantly, β-glucan supplementation prevented the *T. gondii* Wh6 infection-induced impairment to neurite and synaptic ultrastructure. In line with these results, we observed that *T. gondii* Wh6 infection decreased the expression of SYN and PSD-95 in the PFC of mice, consistent with the results of previous studies with other *T. gondii* strains [5]. However, it should be emphasized that the β-glucan intervention alleviated these pathological changes induced by *T. gondii* Wh6 (Fig. 3f, g). Therefore, these findings suggest that *T. gondii* Wh6 infection impaired neuronal integrity and synaptic ultrastructure, and these could be evidently prevented by β-glucan, indicating a neuroprotective effect of β-glucan.

Neuroinflammation, characterized by the activation of microglia and astrocytes, is considered to be a key driver of the progression of neurodegenerative diseases [39, 56]. Previous studies have reported that chronic *T. gondii* infection induces microglial activation and astrogliosis [57, 58]. Our results also showed that chronic *T. gondii* Wh6 infection causes the activation of microglia and astrocytes in the PFC of mice (Figs. 4, 5), indicating that massive neuroinflammation was caused by *T. gondii* Wh6. Importantly, β-glucan administration attenuated the hyperactivation of microglia and astrocytes induced by *T. gondii* Wh6. In addition, the activation of microglia and astrocytes leads to the release of po-inflammatory cytokines, such as IL-6, TNF-α and IL-1β, which is closely associated with the damage of neurons and neurodegeneration [59, 60]. Our findings also confirmed that *T. gondii* Wh6 infection upregulated the expression of IL-6, TNF-α and IL-1β in the PFC region (Fig. 6), which was restricted by β-glucan, suggesting that the neuroinflammation and cytotoxicity of hyperactivated glial cells could be reduced by β-glucan. Thus, we speculate that chronic *T. gondii* Wh6 infection causes neuroinflammation, eventually impairing cognitive function, while β-glucan can suppress this process.

**Fig. 7:**
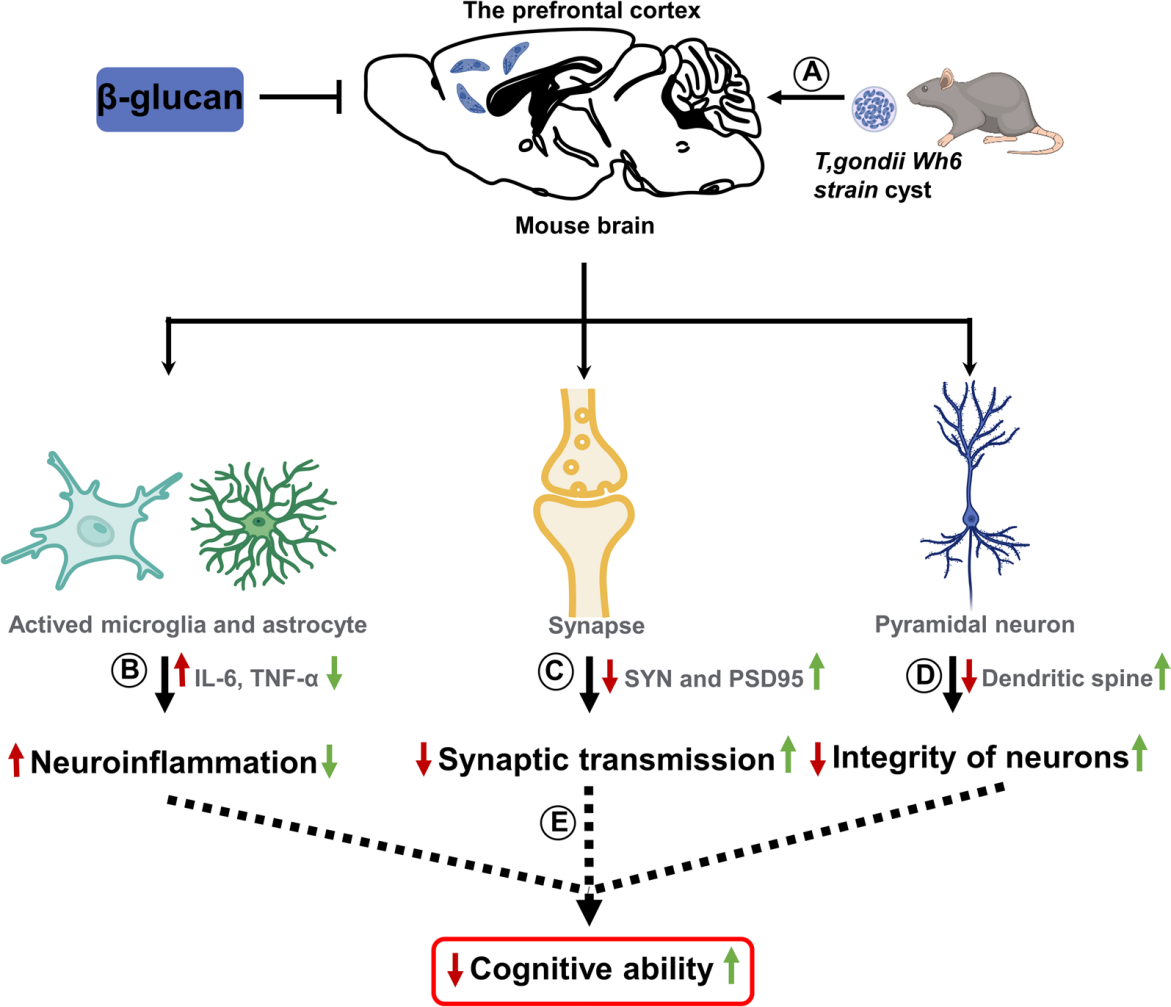
Graphical summary of the protective effect of β-glucan on the cognitive impairment induced by *T. gondii* Wh6. **a** *Toxoplasma gondii* Wh6 tends to infect the prefrontal cortex after passing through the brain. **b** *Toxoplasma gondii* Wh6 induces the activation of microglia and astrocytes and the release of pro-inflammatory cytokines, such as IL-6 and TNF-α, which subsequently exacerbate the neuroinflammation. **c** The presence of *T. gondii* reduced synapse-related proteins, such as SYN and PSD-95, thereby affecting the information transmission capacity of synapses. **d** *Toxoplasma gondii* Wh6 affects pyramidal neuron integrity by impairing dendritic spine density. **e** Taking all elements together, the decline of cognitive function is induced. Red arrows represent events induced by *T. gondii* infection; green arrows represent the amelioration by β-glucan adminstration

β-Glucan, the major bioactive component extracted from mushrooms and fungi*,* has been shown to promote immune response and induce the expression of inflammatory cytokines at a low dose [61]. It has been reported that β-glucan extracted from *L. donovani* can inhibit acute *T. gondii* infection [19]. Recently, several studies, including one from our group, demonstrated that dietary β-glucan can improve cognitive impairment via remolding of gut microbiota [23, 24]. Importantly, consumption of a diet rich in β-glucan (polysaccharide) has been reported to improve cognitive performance in humans, thereby reducing the incidence of cognitive impairment [62]. In the present study, we found that intraperitoneal injection of β-glucan could maintain the structural integrity of neurons and the complexity of dendritic spines, improve synaptic ultrastructure damage and inhibit neuroinflammation, thereby preventing goal-directed behavioral impairment induced by chronic *T. gondii* Wh6 infection (Fig. 7). To our knowledge, the treatment strategy for the *T. gondii*-induced cognitive impairment is still in the clinically shameful situation of having no effective treatment drugs [63]. The present study suggests that β-glucan may be a potential drug for the treatment of cognitive diseases caused by *T. gondii* infection.

## Conclusions

The present study demonstrates that chronic *T. gondii* Wh6 infection causes goal-directed behavioral impairment in mice and that this impairment could be prevented by intraperitoneal administration of β-glucan. Our findings suggest that *T. gondii* Wh6 strain, similar to other strains, is a risk factor for neurodegenerative diseases. Importantly, β-glucan may be an effective drug candidate for the treatment of psycho-behavioral disorders induced by toxoplasmosis.

## Data Availability

All data generated or analyzed during the research process are included in this published article.

## Abbreviations

AD: Alzheimer's disease
CNS: Central nervous system
IL: Interleukin
PBS: Phosphate buffer saline
PD: Parkinson's disease
PFC: Prefrontal cortex
PSD: Postsynaptic density
RT-PCR: Real-time reverse transcription PCR
SDS-PAGE: Sodium dodecyl sulfate–polyacrylamide gel electrophoresis
SYN: Synaptophysin
TNF-α: Tumor necrosis factor alpha
TOM: Temporal order memory
